# Central Retinal Artery Occlusion in the Emergency Department: A Case Report of Prompt Diagnosis and Treatment

**DOI:** 10.7759/cureus.96423

**Published:** 2025-11-09

**Authors:** Gudithi Bhuvaneswari, Salah Ali AlGhanem, Abdulla Ali Dawaishan

**Affiliations:** 1 Emergency Medicine, King Hamad University Hospital, Busaiteen, BHR; 2 Ophthalmology, King Hamad University Hospital, Busaiteen, BHR

**Keywords:** anterior chamber paracentesis, canadian triage and acuity scale, central retinal artery occlusion, hyperbaric oxygen therapy, ocular massage, sudden painless loss of vision

## Abstract

Central retinal artery occlusion (CRAO) is an ocular emergency, and it is equivalent to a stroke in the eye. Hyperbaric oxygen therapy (HBOT) has been shown to result in visual improvement in CRAO. It maintains oxygenation of the retina by choroidal blood supply, thus decreasing edema, and preserves tissue that is compromised adjacent to the ischemic area. Compared to traditional treatment regimens for CRAO, such as ocular massage, anterior chamber paracentesis, and thrombolysis, which have a risk of hemorrhage, HBOT is less invasive and has a better visual outcome.

We present a case of a middle-aged female who presented with sudden, painless loss of vision and was triaged as category 4 as per the Canadian Triage and Acuity scale by a triage nurse and diagnosed as an ophthalmic emergency, CRAO. Early HBOT has improved the visual outcome in follow-up visits after three months.

## Introduction

The incidence of central retinal artery occlusion (CRAO) is about 1-1.9/100,000 people in the United States, with less than 2% involving both eyes [1,2]. The age-standardized incidence is 2.7/100,000 person-year in Germany, 2.53/100,000 person-year in Japan, and 1.8/100,000 person-year in Korea [3,4]. The incidence increases with age, with around 10/100,000 person-years in those aged 80 or older [5]. CRAO is most common in individuals aged 60 to 70 [6]. In the pediatric population, CRAO occurs mostly due to non-atherosclerotic causes like embolism from calcified cardiac valves, trauma, and infection. Males are more affected than females.

## Case presentation

A 52-year-old female with no known comorbidities came walking to the ER with chief complaints of decreased vision in her right eye for 1 day after waking up from sleep and complete loss of vision for 3 hours. She was triaged as category 4 according to the Canadian Triage and Acuity Scale by the triage nurse. The patient had a history of transient loss of vision in the right eye for the past week.

On arrival, her triage vitals were blood pressure (BP) 148/98 mmHg, pulse rate (PR) 115/min, respiratory rate (RR) 20/min, and peripheral oxygen saturation (SPO2) 97% on room air. Systemic examination was not significant.

**Figure 1:**
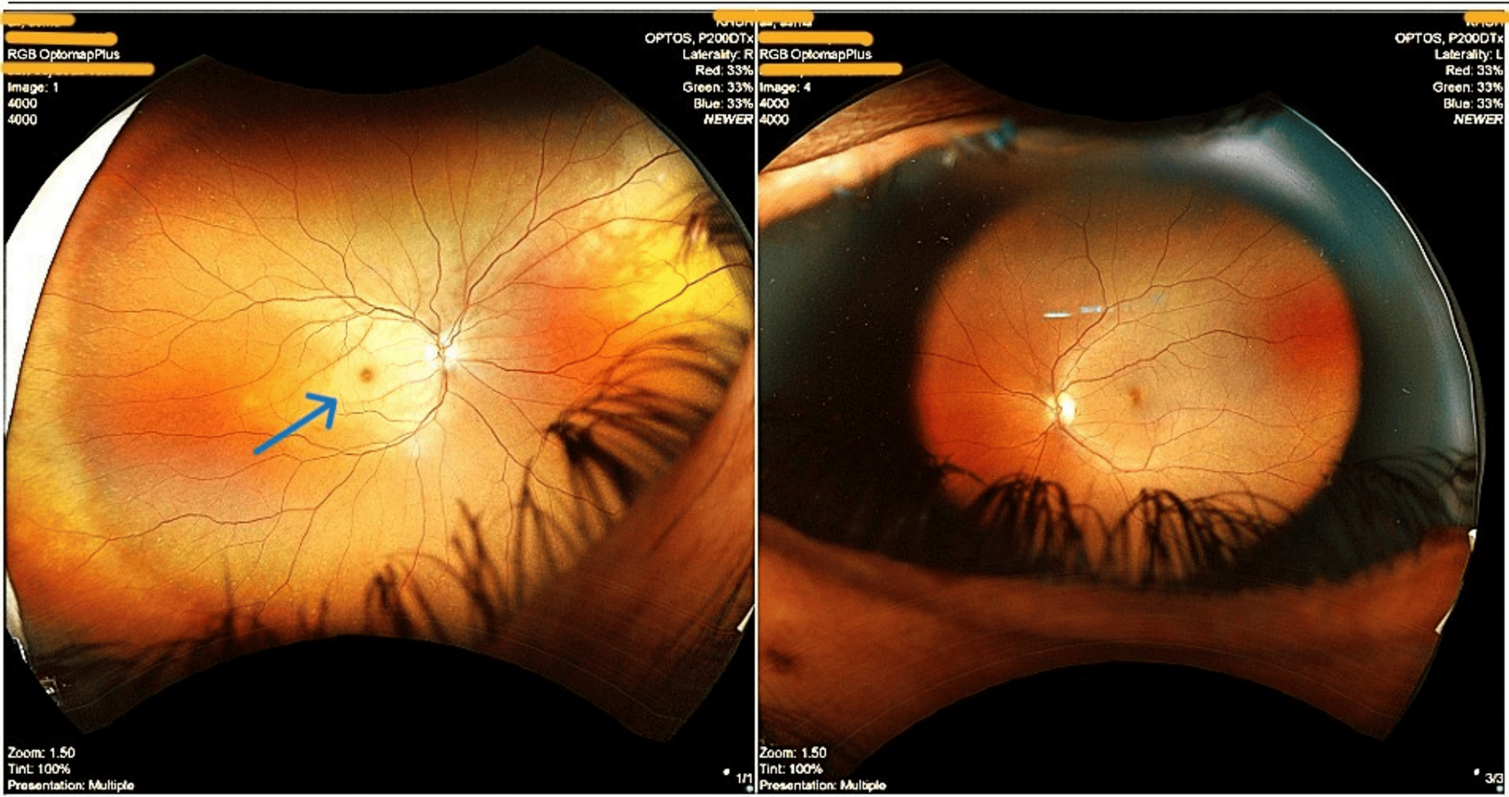
Fundoscopic examination of the right eye showed a pale macula with multiple emboli in the retinal arterioles, mostly inferiorly The blue arrow shows the pale macula in the right eye.

On ophthalmic examination, visual acuity was perception of light in the right eye and 6/9 in the left eye. On the swinging flashlight test, relative afferent pupillary defect (RAPD) was noticed in the right eye. Fundoscopic examination (Figure 1) of the right eye showed a pale macula with multiple emboli in retinal arterioles, mostly inferiorly, and was diagnosed as central retinal artery occlusion. The patient was taken for urgent anterior chamber paracentesis, but she refused and was managed conservatively by topical intraocular pressure (IOP)-lowering drugs like Brinzolamide and Timolol ophthalmic drops, ocular massage, and low-dose oral antiplatelet Aspirin 75mg was administered stat. No significant improvement in vision was seen.

**Figure 2:**
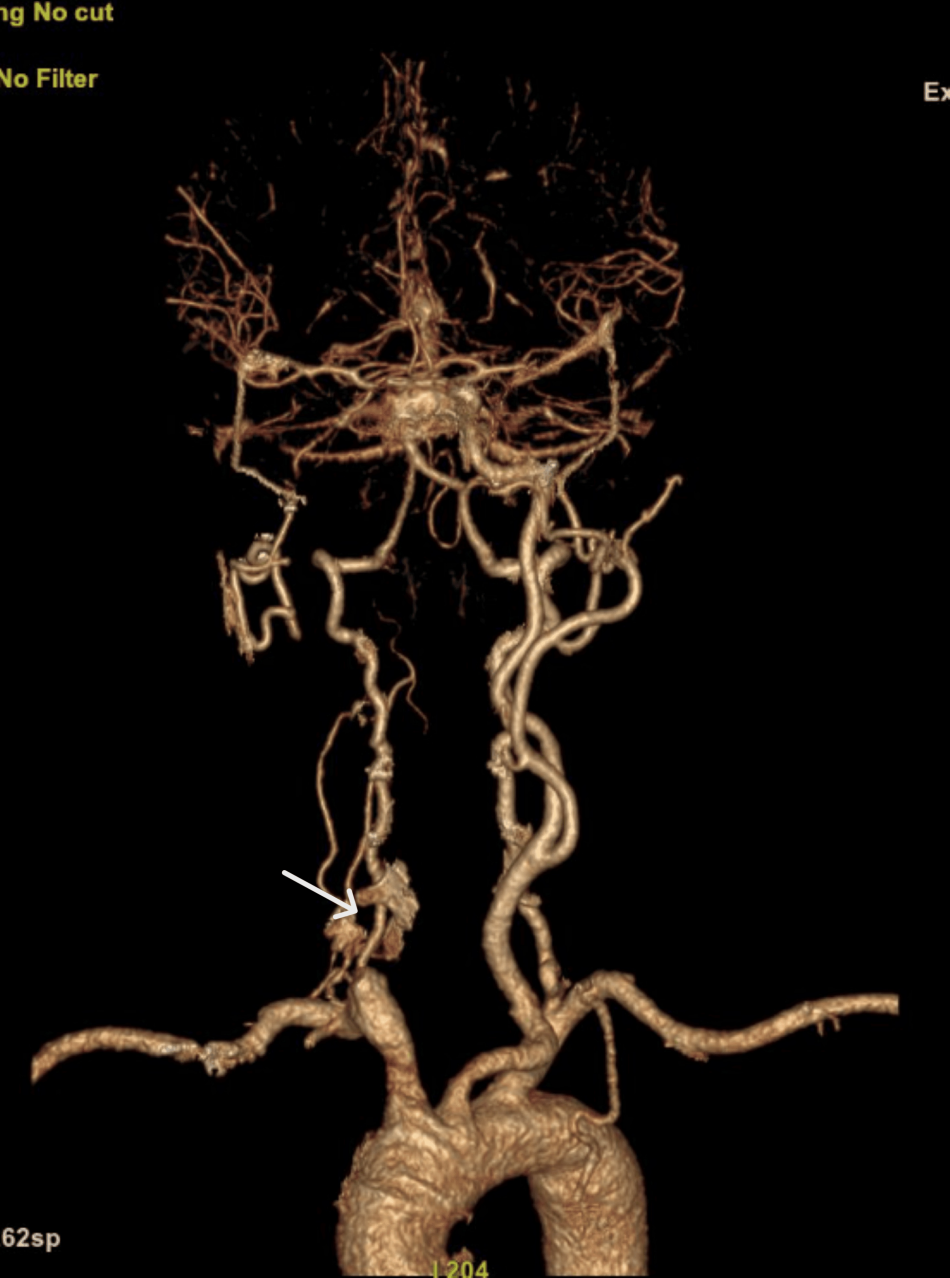
CT brain with angiogram showed complete occlusion of most of the right CCA, ICA (C1 to C6 segments), and ECA. Bilateral ACA and MCA are patent and show normal contrast flow. Posterior circulation, including bilateral vertebral arteries, is patent. The white arrow shows complete occlusion of the right CCA. CCA: common carotid artery; ICA: internal carotid artery; ECA: external carotid artery; ACA: anterior cerebral artery; MCA: middle cerebral artery

An urgent CT angiogram of the brain was done. CT brain with angiogram (Figure 2) showed complete occlusion of most of the right common carotid artery (CCA), internal carotid artery (ICA) (C1 to C6 segments), and external carotid artery (ECA). The bilateral anterior cerebral artery (ACA) and middle cerebral artery (MCA) were patent and showed normal contrast flow. Posterior circulation, including bilateral vertebral arteries, was patent.

Her labs showed red blood cells (RBC) 7.34/mm3, hemoglobin (Hb) 17.2g/dL, and hematocrit (HCT) 53.6. The electrocardiogram (ECG) was normal. As the patient was not willing to undergo any invasive procedures like thrombolysis and anterior chamber (AC) paracentesis, she was taken for emergency hyperbaric oxygen therapy (HBOT) almost seven hours after symptom onset. After one session of HBOT, the patient's vision improved from perception of light to hand movements.

During the hospital stay, she was evaluated for arteritic causes of CRAO; her homocysteine levels were within the normal range: C-reactive protein (CRP) 12 mg/L, erythrocyte sedimentation rate (ESR) 63 mm/hr (elevated), normal rheumatology profile, and glycated hemoglobin (HbA1c) 6%. However, she was not evaluated for polycythemia, as she was not willing to undergo invasive procedures. The probable cause of CRAO is thought to be non-arteritic by thrombus, and the right CCA as the source of thrombus. The predisposing factor in view of the raised RBC, hemoglobin, and hematocrit was considered to be polycythemia vera; however, a confirmatory diagnosis was not made.

Repeat CT brain with angiogram after two days showed no significant changes. There was total occlusion of the right common iliac artery with a long segment right ICA occlusion from origin up to the intracranial C6 segment, with sparing of the C7/terminal segment. Bilateral ACA and MCA were patent and showed normal contrast flow. Posterior circulation, including bilateral vertebral arteries, was patent. The right ECA showed a lack of contrast enhancement suggestive of complete occlusion. The left CCA, ICA, and ECA were patent.

She was discharged on the second day of admission and was followed up for HBOT. A total of eight sessions of HBOT were given. Follow-up visual acuity after 6 months was 6/18 in the affected eye.

## Discussion

CRAO is an ophthalmic emergency characterized by the sudden blockage of the central retinal artery, the primary blood vessel that supplies the retina. It leads to sudden and severe painless loss of vision in the affected eye. The American Heart Association included CRAO in the definition of acute ischemic stroke. Acute ischemic stroke is defined as an episode of neurological dysfunction caused by focal cerebral, spinal, or retinal infarction by the American Heart Association [1].

Hayreh and Zimmerman classification of CRAO

Non-arteritic CRAO

This is the most common and is seen in 66% of cases of CRAO. It is due to the permanent occlusion of the central retinal artery.

Non-arteritic CRAO With Cilioretinal Artery Sparing

This is seen in 13% of cases.

Transient Non-arteritic CRAO

This is seen in 16% of cases. It is due to the temporary blockage of the central retinal artery, which lasts from minutes to hours. Causes include transient severe fall in perfusion pressure of the central retinal artery, a migrating embolus, and increased intraocular pressure.

Arteritic CRAO

This is seen in 5% of cases and is due to central retinal artery and posterior ciliary artery occlusion. The most common cause is giant cell arteritis [7,8].

Pathophysiology

The blood supply of the retina is mainly from two vascular systems: the central retinal artery and the choriocapillaries. The inner retina is supplied by the central retinal artery, and the outer retina and retinal pigment epithelium are supplied by the choriocapillaries. The choriocapillaries are further supplied by the short posterior ciliary arteries. So when there is occlusion of the central retinal artery, the inner retina is mainly affected. The nerve fiber layer and ganglion cell layer of the inner retina become ischemic and lose transparency.

In cases of incomplete occlusion of the central retinal artery, there will be reversible loss of vision, and vision can be regained after 8-24 hours. There is macular collateral circulation from the cilioretinal artery in about 25% of individuals [9]. Those with this anatomical variant may have good prognoses and less severe presentations.

Assessment

CRAO presents with sudden, painless monocular loss of vision, which occurs over seconds. A history of transient loss of vision, also known as amaurosis fugax, may also be present. Atherosclerotic disease is one of the risk factors.

On swinging flashlight examination, a RAPD in the affected eye, also known as Marcus Gunn pupil, was noted. Intraocular pressures, anterior chamber examinations, and extraocular eye movements should be done. A detailed neurologic examination should be done for focal neurologic deficits. On fundoscopic examination, a diffusely pale retina with a cherry-red macular spot was found. A cherry-red spot may not always be present in some cases of CRAO. In older patients with a history of polymyalgia rheumatica, jaw claudication, and scalp tenderness, arteritic CRAO is suspected.

Ipsilateral carotid artery disease or occlusion can cause contralateral motor or sensory deficits. So the carotid pulse should be checked on both sides.

Diagnosis

CRAO is a cerebrovascular accident analogue and should be evaluated like a stroke or transient ischemic attack. The ophthalmic workup includes color fundoscopy and an optical coherence tomography (OCT) of the macula.

Color fundoscopy demonstrates retinal whitening in the affected eye as compared to the normal eye. A cherry-red macular spot may or may not be present. OCT demonstrates hyperreflectivity of the inner retinal layer in acute phases. However, some cases may have hyperreflectivity of the inner nuclear layer, known as paracentral acute middle maculopathy. In late phases, thinning of the inner retina is seen.

A fundus fluorescein angiogram (FFA) shows slow arterial filling of the dye. In non-arteritic CRAO, the choroid fills normally, whereas there will be absent or late filling of the retinal arterioles. In arteritic CRAO or ophthalmic artery occlusion, the choroidal phase may show hypoperfusion. FFA may be normal in a few cases of CRAO. However, FFA is not diagnostic for CRAO.

Lab investigations include the complete blood picture, random blood sugar, and coagulation profile. An ESR and CRP to rule out giant cell arteritis.

If symptom onset is less than 4.5 hours, a CT brain should be done to rule out intracranial hemorrhage, and thrombolytic therapy must be considered [1]. Other diagnostic tests include HbA1c, lipid profile, antinuclear antibody, Rh factor (rheumatoid factor), fluorescent treponemal antibody absorption test, hypercoagulability labs, carotid artery imaging, electrocardiogram, echocardiogram, outpatient Holter monitoring, carotid duplex ultrasound, CT, magnetic resonance, and digital subtraction angiography.

Treatment

The visual prognosis of CRAO remains poor despite treatment, which includes the following.

- An immediate ocular massage

- Intraocular pressure-lowering agents: IV Acetazolamide 500 mg, IV Mannitol (1.5 - 2 g/kg over 30 min to 1 hour), and topical Timolol (0.5%, 1 drop q12th hourly)

- IV thrombolysis (fibrinolysis): The American Heart Association recommends considering IV thrombolysis in cases of non-arteritic CRAO if symptom onset is within 4.5 hours, if there are no systemic contraindications to IV thrombolysis [10]. The IV infusion of alteplase 0.9 mg/kg

- Intra-arterial thrombolysis: In patients who are not candidates for intravenous thrombolysis, presenting within 6 hours of symptom onset, intra-arterial thrombolysis is considered if the facility is available in health centers [1,11]. Adverse effects are dislodgment of atheromatous plaque, arterial dissection, and spasms

- HBOT: The main purpose of HBOT is to increase blood oxygen tension. It mainly acts by hyperoxygenation and reduction of gas bubble size in the bloodstream. The air we breathe contains about 21% oxygen. In HBOT, 100% of oxygen is delivered under atmospheric pressure that is 2-3-fold greater than normal atmospheric pressure at sea level. Thus, the arterial oxygen pressure and tissue oxygen pressure rise, and this increase in oxygen tension will have favorable impacts on biochemical, cellular, and other physiological processes in the body. Also, the higher oxygen in the tissues allows the damaged cells to heal by themselves more effectively. Under high atmospheric pressures in a closed chamber, there will be increased solubility of oxygen in blood plasma, which is crucial for oxygen requirements in the body, especially for ocular tissues like the retina, vitreous body, and choroidal vasculature. Administering about 2-2.5 atm over 90 minutes within 8 hours of the onset of symptoms is helpful in CRAO [12]

- Pentoxifylline: Pentoxifylline increases the retinal blood flow. However, the drug has many limitations for use in patients with CRAO [13,14]; sublingual isosorbide dinitrate: isosorbide dinitrate causes vasodilatation and thus increases retinal blood flow. The recommended dose is 10 mg sublingual.

- Supplemental oxygen

- IV methylprednisolone: Steroids are mainly useful in arteritic CRAO, especially caused by giant cell arteritis. The dosage is 1 g per day for 1-3 days.

- Neodymium: yttrium aluminum garnet (Nd: YAG) laser embolectomy: A meta-analysis noted that although visual recovery is good, in most cases, vitreous hemorrhage is frequent [15,16].

- Pars plana vitrectomy: Thrombus removal by incision over an ateriole has been reported in a few cases [14,16,17].

- Anterior chamber paracentesis: It is an invasive procedure, and there is a risk of ocular injury and infection. So, authors recommend against its use in CRAO [18].

- Carbogen: Carbogen inhalation combined with anterior chamber paracentesis showed no benefit in CRAO compared to no interventions [19].

- Hyperventilation paper bag method: Hyperventilation into a paper bag can induce respiratory acidosis due to CO2 rebreathing and vasodilatation.

Differential diagnosis

The differential diagnosis included ophthalmic artery occlusion, paracentral acute middle maculopathy, Purtscher retinopathy, hypertensive retinopathy, retinitis around the optic disc, commotio retinae, lysosomal storage disorders, and adverse drug reactions.

Prognosis

Despite recent advances in the medical field, the visual prognosis of CRAO is poor, and less than 10-20% of patients regain functional vision [10].

CRAO is a stroke analog, and similar to acute ischemic stroke, it is associated with an increased risk of recurrent vascular events [20].

## Conclusions

Middle-aged females presenting to the ER with similar complaints multiple times are often undertriaged and missed. Complete examination and evaluation are required for every visit, and each complaint should be taken seriously until they were ruled out for organic and metabolic causes. The above patient was treated with early HBOT, and her vision improved from perception of light to 6/18 in follow-up visits after three months. HBOT should be considered more in the treatment of CRAO, which is less invasive. Early HBOT improves vision. However, more studies are required for the exact time window to start HBOT in CRAO.
